# Deterioration in mental health: towards a conceptualization based on patients’ perspectives

**DOI:** 10.1080/17482631.2026.2644587

**Published:** 2026-03-16

**Authors:** Janne Låver, Andrew Athan McAleavey, Irene Valaker, Janne-Merete Torset Øien, Christian Moltu

**Affiliations:** a Department of Health and Caring Sciences, Western Norway University of Applied Sciences, Førde, Norway; b District General Hospital of Førde, Førde, Norway

**Keywords:** Deterioration, negative outcomes, treatment failure, MBC, qualitative research methods

## Abstract

**Purpose:**

This study explores the concept of deterioration from the perspective of patients with mental-health problems, aiming to increase our understanding of this complex phenomenon.

**Method:**

Participants were 15 patients in an outpatient public mental health setting. Transcribed interviews were analyzed using thematic analysis.

**Results:**

An overarching theme and four subthemes were identified. The main theme (an overwhelming sense of depletion from being in constant vigilance) encompasses experiences with deterioration as an ever-present potential participants had to plan for, and protect themselves from. In subtheme 1 (losing or having one’s perspective changed), participants described increased symptoms, relational problems, and avoidance. In subtheme 2 (being in a state of negative emotional reactivity), participants described feeling brittle and easily negatively affected by life events. Subtheme 3 (experiencing physical and psychological pain), encompasses experiences with increased or newly emerged pain, related to psychological distress. Subtheme 4 (becoming less authentic with oneself and others), details how participants intentionally or unintentionally hide their symptoms and difficulties.

**Conclusion:**

Mental health deterioration is a multifaceted concept that includes, but is not limited to symptomatic increases. Patients’ overall life functioning and the fact that patients may choose to hide their symptoms must also be taken into account.

## Clinical Impact Statement

**Question:** How do patients in treatment for mental health problems describe the experience of deterioration?

**Findings:** Deterioration of mental health affects every aspect of patients’ lives, including function and relationships, and cannot be viewed solely as symptom worsening.

**Meaning:** Identifying deterioration solely on the basis of symptom worsening ignores aspects of patients’ experiences and may under-emphasize the importance of this phenomenon.

**Next steps:** The findings highlight an emerging difference between quantitative and qualitative research in conceptualization of deterioration, a discrepancy which must be resolved.

According to one estimate, 5−10% of mental health patients deteriorate during their contact with a treatment provider (Lambert, 2013). Traditionally, research on the concept of patient deterioration has received much less attention than research on treatment success (Halfond et al., 2020; Honkalampi et al., 2025; Krivzov et al., 2021). Lack of awareness of, and knowledge about deterioration might hinder its detection and recognition, making it more challenging to address in treatment. Therefore, we need a better understanding of what constitutes deterioration, and how deterioration manifests in individual patients.

Deterioration has been defined in many ways in the past. It has mostly been studied alongside other related concepts such as negative or adverse outcomes, or treatment failure. Though some studies have used the terms “negative outcome” and “deterioration” interchangeably (e.g., Mohr, 1995), other studies on negative or adverse outcomes report deterioration as one specific aspect of negative outcomes (see e.g., Klatte et al., 2025; Lambert, 2011; Paveltchuk et al., 2022; Schermuly-Haupt et al., 2018). In such studies, deterioration usually refers to symptomatic deterioration in the form of exacerbating or new symptoms.

In many cases, deterioration is even more narrowly defined as a predetermined numerical change in self-reported scale scores, for instance using the Reliable Change Index (RCI) and related definitions of clinically significant change (Honkalampi et al., 2025; Jacobson & Truax, 1991; Rozental et al., 2018; Shimokawa et al., 2010; Østergård et al., 2020). To qualify as “deteriorated” in these operationalizations, patients must have substantially worse symptom severity after treatment compared to before treatment, while patients who fail to improve, improve in some ways while deteriorating in others, or deteriorate in less substantial but still important ways are not identified as deteriorators. This has led several authors to suggest that less conservative criteria be considered for deterioration (McAleavey, 2024; Peipert et al., 2023; Wise, 2004).

Although symptoms are an essential aspect of mental health problems and symptom worsening is necessary to define the construct of deterioration, it might be insufficient as overall symptom reduction may not be feasible, or symptomatic outcomes are not what is most important to patients (Ladmanová et al., 2025; Moltu et al., 2017). Many patients have reported that their experience of deterioration is not the same as the definitions based on quantitative self-report data (Sales et al., 2018). For instance, despite clinically significant deterioration on symptom measures, patients can still experience therapy as both effective and helpful in alleviating their difficulties (De Smet et al., 2019; Zimmermann et al., 2012). Moreover, what appears to be deterioration on outcome measures could be the result of: (a) treatment ending while the patient was still in a change process (De Smet et al., 2025); (b) patients feeling that they needed something more or something different from treatment (Borjesson & Bostrom, 2020; Cuperfain et al., 2021); or (c) cases of paradoxical outcomes (Stänicke & McLeod, 2021). For example, McElvaney and Timulak (2013) found that patients categorised as having poor outcomes on an outcome measure (CORE-OM) experienced more heightened awareness of problematic functioning compared to those with good outcomes, meaning that the deterioration in scores could be because patients were becoming more in tune with their emotions. This incongruence between measured scores and actual experience suggests that deterioration is defined too narrowly by quantitative outcome scores alone.

Aside from changes in self-report scores, several discrepant and varied experiences have been identified that might be important to include for understanding deterioration. For example, Dimidjian and Hollon edited a special journal issue that focused on treatment failure (Dimidjian & Hollon, 2011). Contributors selected cases based on varying definitions of treatment failure, some of which seemed tied to deterioration. The included cases defined treatment failure as, for example, premature termination (Boswell et al., 2011), relapse (Arch & Craske, 2011), lack of engagement with treatment (Brozovich & Heimberg, 2011), and impeded response to treatment (Newman, 2011). Clearly, there are many different ways for a case to be considered a treatment failure, at least some of which may overlap with deterioration.

Concepts such as negative/adverse events encompass deterioration to some extent, but they also contain other elements that seem to be less relevant to deterioration specifically. Increased suicidal ideation or self-harm (Honkalampi et al., 2025; Klatte et al., 2025; Paveltchuk et al., 2022), decreased well-being or increased distress (Curran et al., 2019; Schermuly-Haupt et al., 2018), or loss of motivation or hope (Vybíral et al., 2024), can be seen as specific expressions of deterioration. Related concepts, such as non-improvement and premature termination (Bystedt et al., 2014; Lindegaard et al., 2024), lack of flexibility in treatment (Paveltchuk et al., 2022), lack of goal attainment (Knox et al., 2023), experiencing loss of control, and less willingness to self-disclose (Vybíral et al., 2024), may also be considered deterioration in some cases. In summary, quite a few concepts have been studied that are related to deterioration, and the specific meaning of deterioration may vary from study to study.

Scientific knowledge about common features of deterioration should be helpful for the field of psychotherapy, and exploring first-hand experiences can be beneficial for understanding complex phenomenon, such as deterioration. Patients’ negative experiences of psychotherapy have been explored in research (see, e.g., Vybíral et al., 2024). However, the reviewed studies focus on many different negative experiences, some of which could contribute to deterioration (such as therapists’ misbehaviour), but they do not study deterioration in and of itself. Deterioration can be a direct consequence of treatment processes or be associated with life events completely unrelated to treatment. Any analysis that prioritises the context of treatment rather than the broader patient experience risks limiting findings that are essential to form a qualitative understanding of deterioration. If treatment is the organising focus of a study, then the full narrative of the patient can slip into the background (Moltu et al., 2023). Thus, the aim of this study was to explore the experience of deterioration from the first-person perspective of patients with mental health problems. Our specific research question was: Which experiences do patients draw on when describing deterioration?

## Methods

### Design

We position ourselves within the epistemological orientation of social constructionism (Berger & Luckmann, 1991), and apply an inductive qualitative approach for this study due to its exploratory research question, as qualitative studies are particularly suited for gaining knowledge into underexplored clinical processes (McLeod et al., 2021). In social constructionism knowledge is viewed as created through social processes and interactions. Meaning is constructed based on our knowledge-base, preconceptions, context and social realities. As the goal of our study was to gain in-depth knowledge into the experience of mental health deterioration, exploring first-hand experiences was considered crucial (Natvik & Moltu, 2016). Data were collected through individual in-depth interviews with patients, and analysed using reflexive thematic analysis (Braun & Clarke, 2022).

### Recruitment

Participants were recruited from autumn 2022 to autumn 2024 from three outpatient clinics at separate locations within the geographical area of a hospital region in Western Norway. The government funded clinics served a general mental health and substance abuse population at a secondary specialist care level.

We aimed to recruit patients in active treatment for primary psychiatric problems or primary substance abuse problems in outpatient care. The inclusion criteria were: (1) age >18 years; (2) treated with psychotherapy at time of recruitment; and (3) speaking a Scandinavian language or English. We excluded patients with active psychosis. Patients were informed of the study and recruited by either 1) Information posters in the clinic’s waiting area, or information leaflets handed out by their therapists. Prospective participants either contacted the first author themselves, or gave consent for their therapist to convey their contact information; or (2) by answering a questionnaire in a corresponding research project (Ki et al., 2025) in which patients gave consent to being contacted for an interview. Patients were consequently contacted by a researcher to schedule an interview.

### Participant characteristics

Written informed consent was obtained from all participants. The total sample consisted of 15 patients. We describe the group of participants generally in aggregated form to protect their anonymity. Participants ranged in age from their 20 s to 60 s, nine were female and six were male (We did not ask them specifically about their gender or orientation.). Participants were predominantly White Scandinavian, which is typical of the population this hospital region serves. All but one participant was in active psychotherapeutic treatment at the time of the interview, whereas one participant had just finished therapy. Participants had to have a diagnosed mental health or substance use disorder with substantial loss of function to be eligible for treatment at these clinics. We did not specifically ask participants what their diagnosis was; instead, we asked them to describe why they were in treatment. Participants described problems such as mood swings or negative mood, social isolation, feeling anxious, being easily overstimulated, difficulties with sorting and processing emotions, suicidal ideation, self-harm, dissociation, and manic episodes. None of the participants described experiences with psychotic episodes, and none reported primary addiction problems. Participants’ treatment experiences varied in length and number of episodes, and most of them had been in treatment more than once.

### Data generation

Mental health issues can be considered sensitive interview topics as they are deeply personal and vulnerable experiences. Therefore, we decided to conduct individual interviews to provide a safe space where participants would feel comfortable with being open when sharing their stories. Physical face-to-face interviews can help to create a comfortable atmosphere and facilitate immediate responding to distressing emotions that may arise during or right after the interview (Westland et al., 2024). Interviews were conducted between 2022 and 2024 by JL or JMTØ. Both interviewers were nurses with broad experience meeting patients in sensitive situations. All but one of the included participants were in active treatment at the time of the interview, and all had the opportunity to discuss any difficulties that might have arisen from participation with their respective therapists. All interviews were conducted either at the interviewers’ workplace, which is located at the hospital, or at the mental health clinic. When interviews were conducted at the interviewers’ workplace, the last author (a clinical psychologist) was available if needed for screening patients before the interviews were conducted, and for debriefing the participant and interviewer after the interviews. No such need arose during the interview process.

We conducted semi-structured interviews based on a topic guide covering four broad themes. All interviews addressed these topics in relation to deterioration: Experiences with living with a mental health problem, experiences with mental health and social relationships, experiences with mental health and work, and experiences with how mental health problems affected participants’ daily lives. The interview guide was piloted with one patient who fit the inclusion criteria, and minor adjustments were made to it. After careful consideration of the material, and obtaining informed consent, we decided to include the pilot interview in the data material. Interviews lasted between 36 and 94 minutes (mode ca. 70 minutes), and were transcribed verbatim. The transcribed interviews constituted the data material for analysis.

### Data analysis

The data were analysed using reflexive thematic analysis (Braun & Clarke, 2022), and codes were created inductively. Analysis was an inherently iterative process, characterised by continuous engagement with the data, and constant comparison was employed throughout. Below, we outline the phases of the analytic process, conducted to support reflexive engagement and interrogate interpretations when co-constructing meaning within the data.

1. Process of familiarisation: The first author read and re-read all the interviews to become familiarised with the content. JL then selected those parts of the interviews that were relevant to the research question, and transferred these to a separate document. AAM, JMTØ, and CM read these interview excerpts and met with JL for an initial discussion and a preliminary understanding of the content.
2. Building on the ideas from the initial discussion, JL read all the material again, and identified meaning units. These meaning units were sorted into five preliminary themes. For example, the meaning unit “I”m really good at ignoring things, really, really good at it. My condition might be quite a bit worse, but I’m ignoring, ignoring, ignoring, ignoring everything to just get away from the situation,” was tagged with the codes “ignoring symptoms, hiding from self,” and sorted under the preliminary theme “intentionally hiding symptoms.” The research team met again to discuss the preliminary themes and adjustments were made.
3. JL went back and coded all the meaning units, moving and reconsidering them for thematic consistency, and assessing the need for changes in the thematic structure such as development of new themes, or removal of unnecessary themes. During this process, one of the preliminary themes, “expected deterioration” was deemed redundant, and the meaning units sorted here were moved to the other themes.
4. When all meaning units had been coded and sorted, JL and CM met for a new discussion. Here, it was decided that the material seemed to fit into an overarching theme, “an overwhelming sense of depletion from being in constant vigilance,” with subthemes.
5. The authors involved in the initial discussion met again to discuss the full analysis and adjust themes and theme names to capture the essence of the content.
6. The full analysis was given to IV for an audit. IV had not been part of the analysis process, and thus, could review the themes and content for consistency and clarity.
7. After receiving feedback from IV, JL made final adjustments to the analysis.

### Ethical considerations

The study was approved by national legislation by the Regional Ethics Committee for Health Research in Norway (Ref: 2021/197005). Participation was based on fully informed written consent, including information about the storage of the collected data and participants’ right to withdraw from the study at any time.

### Research team and reflexivity

JL is a White, female-identifying, PhD candidate in health science with a background in nursing. AAM is a White, male-identifying, clinical psychologist with extensive experience in research on routine outcome monitoring and prediction modelling. IV is a White, female-identifying, associate professor in nursing and health science. JMTØ is a White, female identifying, PhD student in health science. CM is a White, male-identifying, clinical psychologist and professor of clinical psychology with extensive experience in research on health services and psychotherapy. JL, IV, and JMTØ do not have their primary work experience in the mental health field. As such, their expectations and assumptions of the research topic were based more on previous research and general experience from the wider healthcare system and less on specific personal work experience. AAM and CM have extensive experience conducting individual psychotherapy in mental health organisations in the United States and Norway, respectively. In addition, they have extensive experience in mental health research. The mixed professional composition of the research group was beneficial for the research process because it brought forth new perspectives, and increased reflexivity in the group. Although coming from different professional backgrounds, all the members of the research group shared an interest in person-centred treatment, humanistic health perspectives, service improvement, and qualitative research methods.

### Transparency and openness

This article adheres to the APA Style Journal Article Reporting Standards for Qualitative Research (Levitt et al., 2018).

## Results

While we designed the study to explore patients’ experiences of deterioration in the context of psychotherapy, we immediately found that our participants were not concerned with deterioration in the specific context of treatment. Rather, deterioration was an experience related to their total life situation, not only one particular aspect of it. In engaging reflexively with the dataset in our analysis, we generated a main overarching theme that was consistent throughout all the interviews. This theme had four subthemes.

### Main theme: an overwhelming sense of depletion from being in constant vigilance

In this overarching theme, we seek to convey a central aspect that was at the forefront of our participants’ stories when describing their deterioration experiences. Being in deterioration required so much energy and cognitive capacity that it led to an overwhelming sense of depletion from being in constant vigilance. The knowledge that deterioration might occur could, in itself, extend the period of suffering for the participants. Participants described a sense of brittleness in their lives as they felt perpetually alert, wondering whether they would be able to parry the next upcoming difficulty. In the interviews, participants alternated between describing interconnected experiences with anticipating deterioration, being in deterioration, looking back at deterioration, and avoiding future deterioration, all of which were emotionally taxing states. When talking about their experiences, participants described being acutely aware that even though they may feel stable in the moment, remaining stable demanded continuous investment. They never felt completely safe, as deterioration might be lurking right around the corner, at all times. Put simply, experientially, manifest deterioration and latent or potential deterioration seem intertwined, as the energy needed to keep the potential deterioration in check cannot be used for other, more vital purposes. Furthermore, it seemed like life was balancing on an edge between stability and disruption, and when the overall burden of life increased, the balance could be lost and explicit deterioration would manifest.

For some participants, all of life seemed to circle around finding and developing strategies to avoid being triggered and thus experiencing deterioration. Such strategies could involve shielding oneself from a multitude of stimuli, as well as creating plans to cope with all the imaginable scenarios in life. Consequently, avoiding deterioration was described as overwhelming, depleting the participants of resources, and reducing their resilience. Being in a state of deteriorating mental health was experienced as time-consuming, sometimes even all-consuming for the participants. The specific subthemes describe how it affected their perspective towards themselves and others, determined what they could and could not do in life, what they had to do, their choices, and their planning processes. Being in deterioration, or anticipating the next episode of deterioration, could require all their energy, and being in this state of constant vigilance left the participants completely exhausted.

These experiences of deterioration were manifested in the following subthemes.

### Losing or having one’s perspective changed

This subtheme was prominent in all the interviews. Here, participants described a change in their view of life, their perception of themselves, and their relationship towards others. The changed perspective seemed to be on a continuum of severity and could diminish or expand, depending on the participants’ problem presentation. A diminished perspective was typically described by symptoms such as social isolation, avoidance, loss of feelings of joy or meaning, negative thoughts, downcast mood and mood swings, loss of self-esteem, difficulty performing daily activities, a feeling of being utterly exhausted, and suicidal ideation. An expanded perspective was described as involving changes in mood such as feeling invigorated or experiencing euphoria, carelessness in the way one presents oneself, speaking loudly to engage others in one's own way of thinking, oversharing, irresponsible spending, an inability to concentrate or slow down, and relational difficulties with a partner and others. Common elements of both diminished and expanded perspectives included the inability to perform self-care, difficulty concentrating, overthinking, loss of focus, and forgetfulness.

This change in perspective was described by participants as such experiences as losing control of their emotions, avoiding responsibility, needing to be alone, pulling back from their spouse and/or family, ceasing to engage in activities they usually liked to do, not caring for themselves, such as exhibiting poor eating habits or failing to groom or wash, feeling that they were disliked, feeling that life was not worth living, loss of insight into their condition, ruminating, and being self-critical.

I can feel quite disliked and unwanted when I haven’t been in touch with people for a while. And I feel like I don’t fit into society; things like that. And it’s like… it’s terrible really. I don’t want to live when it’s like that. (P7)

Their perspective seemed to move through different phases along the continuum of severity. The participants described moving away from their baseline, and as they experienced more and more symptoms, they might notice that their perspective changed, by either shrinking or expanding. Having their perspective change could affect participants’ ability to stand up for themselves or seek help. This in turn, could significantly impact the depth of the deterioration process. If this process was not halted, a participant might experience either a depressive crisis filled with suicidal thoughts, or an active manic psychosis necessitating admission to an acute psychiatric unit.

It was my little sister who finally said that this can't go on. I remember very little of it because I wasn't sleeping, I wasn't eating, I was just slowly but surely fading away. And I had my own perceptions about what was what; but by the end, I couldn't distinguish anymore. (...) And if my nieces and nephews hadn't been born, and if it were just my siblings, it wouldn't have been enough. But, I didn't want them to experience that loss, so I agreed to be admitted. (...) After I had been admitted for about a month, I spoke to my husband on the phone, and he said: It's so good to talk to you again. You haven't been here. It's like, it wasn't you, it was just a shell. (P6)

### Being in a state of negative emotional reactivity

In this subtheme, participants described a state of heightened sensitivity in which even minimal stimuli, ordinarily innocuous, provoked significant emotional responses. They expressed this in a sense of being “skinless,” a feeling that they more readily absorbed or reacted to stimuli, and were more susceptible to being hurt or wounded. This theme was present in all the interviews.

Well, crying is a really clear sign that I’m not feeling well, and it can happen really quickly. It can be due to such tiny things as the coffee being a bit tricky to make that morning; it doesn’t take much at all. (P1)

Some participants discussed how small events could trigger changes in mood, such as feeling negative, becoming anxious and afraid, or being easily irritated or angry.

The psychologist said I should try to be aware of what irritates me. What I can do differently so it doesn't irritate me. But, I can't manage that in the moment. Like at work, there was one day when several people were standing in front of the coffee machine talking, and I needed coffee, you know. I didn't say anything, but it affected me for a long time afterward. I feel it as irritation in my body, just move already, you know. And that feeling sticks in my body and snowballs, and I get irritated more and more quickly. It's especially when I'm feeling down. (P9)

Some participants said they were easily overstimulated, even when they were in their habitual state, and that when they were deteriorating, they felt almost “allergic” to stimuli such as sounds, light, and people. One participant even talked about feeling allergic to herself when she was in such a state. It was difficult for participants, in the moment, to articulate why or what specifically triggered a change in their state. However, lack of sleep or feeling exhausted usually contributed to heightening their emotional reactivity. If participants knew what could trigger a change in their state, they often tried to avoid it. For example, one participant said she would only mow the lawn on certain days. Otherwise, she could risk inadvertently meeting the neighbour, who might want to chat with her, and she could not handle that.

### Experiencing physical and psychological pain

Some participants said that they felt pain when they deteriorated. Some of these participants experienced pain as part of their symptoms of comorbid somatic disorders, and that pain increased on occasion when they experienced deterioration of their mental health functioning.

I've had a herniated disc in my back for almost ten years now, and on such days when things aren't going well, I can get a headache. And I've got back pain today. Extra back pain. (...) I've got a bit of a headache from all the impressions, but you know, still have a bit more back pain today than other days; everything in the body is connected. It's not like one part is just sitting there. (P8)

For other participants, pain was a more diffuse concept. That is, they talked about experiencing pain in a physical sense, but it was not really physical pain; it was more like a psychological pain, like a knot in the chest. These feelings of psychological pain could be as intense as feeling a somatic pain.

That anxiety I feel, it gives me really strong feelings of pain in my body at times. It’s like, if I have to be social, then I dread it a lot beforehand, and I really just want to go home because of that pain I feel so strongly. I: Yes, but you feel it physically? P13: Yeah, I feel it in my stomach, but it’s not like it hurts a lot, it’s like it’s physical, but it’s a bit hard to define what it really is, it just tightens up a lot. I: It feels physical? P13: Yeah, but it’s not like the pain you get if someone hits you; it’s more like it feels really painful, both emotionally and physically really. (P13)

In addition, experiencing both physical and psychological pain could be a consequence of experiencing deterioration, and for some of those who experience pain, it could lead to a feeling of being deteriorated.

I get physical pain from the mental stuff. The pain goes away when I'm not having a mental episode. But it comes back every time I get depressed, and I can feel it, so then I have to try to take it easy. (P4)

### Becoming less authentic with oneself and others

Many participants said they hid or minimised their emotional distress, and underreported symptoms when they experienced deterioration. For some, such concealment was part of the pattern of behaviour that they had learned and used from quite a young age. Some concealed their symptoms from their family because they did not want to be a burden to them, or they wanted to shield them from distress. Some participants also reported not wanting their family to ask them so many questions, or to fuss and worry about them.

Being with my family and the ones that are closest to me is a bit challenging to. Because this is something I learned from a very young age. That you have to hide, and not show people how you really feel. It’s not that I’m afraid of my husband, because he’s not dangerous. And I’m not afraid of my kids, but there’s a limit to how much crying I can do. How much time I can spend sitting around being sad. So, in a way, I have to hide it. (P15)

Some participants downplayed or suppressed their symptoms and emotions as a coping mechanism. They refused to allow themselves to feel or acknowledge how they were really doing, because that would mean realising how ill they actually were. A few participants reported that they had ignored their symptoms for such a long time that they had somehow lost connection with themselves and how they were really doing, both physically and mentally, which led to their being hospitalised for serious somatic illness.

Several participants talked about how they had hidden symptoms from their therapist or other healthcare providers.

I: For how long have you been seeing your current therapist? P6: Oh, it’s been a few years. I: And how is your relationship with them? P6: Very good, I trust them. I: Do you tell them how things really are? P6: Yes. I: So, you don't try to underreport to them? P6: Um, well, kind of, but I don't do it on purpose. It’s like I said: I’ve never tried to deceive you guys (the healthcare system), but I’ve tried to convince myself that things are better than they really are.

When participants concealed symptoms it could be because they were not ready to open up in therapy, feeling ashamed, or unsure about the format (e.g., what they were allowed to discuss in therapy), or it could be a component of their learned pattern of hiding. This habit of concealment extended to what participants reported on the Measurement-Based Care (MBC) questionnaire, and whether they completed the MBC prior to sessions. One participant said early in the interview that she was getting better, according to her questionnaire results. However, later in the interview, she admitted that her symptoms fluctuated, and that when she was feeling particularly bad, she did not answer the MBC questionnaires because she was afraid of what her answers might disclose. In contrast, another participant said that it was easier being honest on the MBC because of his habit of hiding symptoms when talking to people when he was feeling particularly low.

## Discussion

In this project, we studied mental health patients’ experiences with deterioration, and in the analysis we generated a main theme, and four subthemes. We found that deterioration was a concept that extended beyond the context of psychotherapy, and suffering was at the forefront of our participants’ stories, regardless of the cause. Deterioration was a term that encompassed a broad range of experiences: from awareness of a potential downturn, through natural and extraordinary fluctuations in mood, to an overwhelming sense of depletion.

The main theme, an overwhelming sense of depletion from being in constant vigilance, encompasses experiences of deterioration as an ever-present potential. Even when in their habitual state, participants had to remain vigilant to plan for and protect themselves from potential deterioration. This is important information for mental health care because even when deterioration is not currently actualised, its potential takes a toll on people’s energy. The first subtheme, loosing or having one’s perspective changed, aligned more closely with a traditional presentation of deterioration. Participants described an increase in symptoms related to their problem presentation. Moreover, they described increased relational problems, and increased avoidance, which also extended to difficulties in asking for help. The inability to ask for help could increase the depth of the deterioration process for the participant.

The second subtheme, being in a state of negative emotional reactivity, is constructed around the participants descriptions of feeling brittle and easily affected by what they perceived as negative life events, such as a small change of plans or the thought of having to talk to people. This emotional reactivity significantly impaired participants’ quality of life and often led to increased avoidance. The third subtheme, experiencing physical and psychological pain, captures participants’ experiences of an increase in pain from their existing somatic pain conditions, as well as experiences of newly emerged pain related to their psychological distress. These pain experiences were completely overwhelming for some participants and led to reduced ability to function in daily life. The last subtheme, becoming less authentic with oneself and others, details how participants intentionally or unintentionally hide their symptoms and difficulties from both significant others and their therapist. This lack of self-disclosure could result in participants’ problems going unrecognised, and could prevent participants from receiving the help they need.

### Deterioration as symptom worsening

An increase in symptoms is one expression of deterioration, and the participants in our study gave many examples of how their existing symptoms worsened when they experienced deterioration. This aligns with a traditional definition of deterioration, which refers to the deterioration of symptoms (Honkalampi et al., 2025; Lambert, 2011; Mohr, 1995). This part of the construct should, in theory, be measurable using standard outcome monitoring instruments. However, assessing deterioration based on symptom change has several limitations, such as the occurrence of paradoxical outcomes—a lack of correspondence between different outcome indicators such as patient self-report symptom measures, patient interviews and clinical observations (Ghelfi, 2021; Stänicke & McLeod, 2021). Additionally, there is the basic finding that patients use self-report measures for a variety of purposes beyond the objective reporting of symptoms, such as initiating in-session discussions, and ensuring they receive the services they want or need (Låver et al., 2024).

Measuring deterioration based on an increase in symptoms means that we need to agree on which symptoms to measure, as well as the timing and context in which to assess them. Most of the studies of deterioration or adverse effects, focus on the context of a specific treatment (Cuijpers et al., 2023). Such studies are apt when assessing which treatment should be offered to whom and in what context. However, their conception of deterioration is often predicated by the target symptoms of the intervention, rather than the patient’s full perspective or well-being. An increase in depressive symptoms is reasonably considered evidence for deterioration in a trial of a treatment for depression, whereas it is weaker evidence (or at least different evidence) in a trial that has a different focus.

Given the disconnect between symptomatic worsening and negative outcomes, defining deterioration only as symptom worsening is insufficient. Instead, symptoms must be assessed both individually and in context. Categorising deterioration according to standardised calculations of clinical significance, without taking the patients’ perspective into account, involves risks that signs of deterioration could be overlooked. This can lead to an underestimation of patients' actual deterioration rates (McAleavey, 2021).

### Symptoms and functioning do not go hand in hand

The results of our study showed that even though changes in specific symptoms were important to our participants, other problems were a prominent part of their experiences of deterioration, including negative effects on life functioning, reduced quality of life, and loss of relational safety. This coincides with the findings of Sales et al. (2018) that work-related and relational problems were the problems patients most often wanted help for, which were not captured by standardised outcome measures. Mental health deterioration can prevent patients from living a cohesive life by reducing their ability to balance their problems with work and other social or practical demands in life (Binder et al., 2010; Moltu et al., 2017). As there is evidence that symptomatic outcomes and functional outcomes are not always related, a decrease in symptom severity does not necessarily lead to better overall life functioning. For example, McKnight and Kashdan (2009) investigated the relationships between symptomatic and functional outcomes for depression and found that there were only moderate correlations for symptomatic outcomes and social, occupational and physical functioning. Also, functioning seemed to be less responsive to, and slower to change with treatment. From psychosis research, Best et al. (2020) found that functional and personal recovery were related but had differential relationships with symptomatology. For example, emotional symptoms predicted personal recovery but not functional recovery, and positive symptoms measured at a global level predicted neither form of recovery. In their systematic review of outcome measures used in RCTs for bipolar and unipolar disease, Veal et al. (2024) found that 9% of outcomes previously identified as important to patients were not covered by the 25 most frequently used outcome measures. For example the concept of mental pain, the third most common domain mentioned by patients (Chevance et al., 2020), was not assessed by any of the outcome measures used in the included RCTs. The most frequently used measure, The Hamilton Depression Rating Scale, covered only 59% of outcomes important to patients. Lastly, Ladmanová et al. (2025), identified ten distinct clusters of psychotherapy outcomes identified as important by clients, and found that only one in ten was directly linked to symptoms and problem change. The remaining outcomes were related to functioning, general wellbeing, self-awareness and coping, and outcomes unrelated to psychotherapy such as change in work-situation.

Our participants’ experiences indicate that deterioration is not a constant state. It was a condition they glided in and out of and experienced more or less in their lives. Many of our participants described a sense of alertness and a constantly heightened awareness that deterioration could occur. This meant they had to spend a significant amount of time to plan and implement measures to prevent deterioration from occurring. Consequently, experiencing deterioration significantly affected their overall life functioning, and functional outcomes are relevant measures for assessing deterioration.

### Deterioration in treatment is expected?

Studies of therapists’ experiences with patient deterioration or negative change in treatment show that many therapists see symptom worsening as a natural/intrinsic component of treatment. Some even think that symptom increase is necessary for patients to move forward in therapy (Bystedt et al., 2014; Lindegaard et al., 2024). Although previous research shows that some patients share the view that symptom increase is necessary to achieve a positive treatment effect (De Smet et al., 2025), we found few examples of this perspective in our own analysis. Mostly, participants discussed deterioration as being separate from treatment. A few participants talked about how treatment brought their problems into the foreground, and that this led to an increase in their painful emotional reactivity. Such adverse treatment reactions or side-effects are commonly reported in the literature (see e.g., Schermuly-Haupt et al., 2018), and might mean, but does not necessarily have to mean, that actual deterioration must be concluded. For our participants, they felt it was difficult to single out what was related to treatment, because everything in their lives felt overwhelming.

Understanding the timing of symptoms worsening is essential for assessing deterioration. It is reasonable to expect that some patients may feel worse during certain stages of their treatment, such as exposure treatment for distressing stimuli. However, it might still be important for therapists to address these signs of deterioration in their patients, as attuned therapists understand that these experiences can lead to significant distress (Kleiven et al., 2023). Additionally, therapists should prepare patients for potential side effects of treatment and provide support during these episodes (Kleiven et al., 2023). If episodes of deterioration are to be used therapeutically, it is vital that patients recognise that it can lead to improvement and help them move forward in therapy. Otherwise, there is a risk that patients may disengage from treatment, resulting in a negative experience that deters them from seeking help in the future, as shown by Knox et al. (2023) in their study on clients’ perspectives on psychotherapy failure.

### Concealing symptoms—a symptom in itself?

From the start of this discussion, we have underscored how participants did not organise their experiences of deterioration as reactive to, or as key part of, treatment. Rather, the experiences were an integral part of their daily life. In line with this, when it comes to the finding of increasing inauthenticity, the cornerstone of this phenomenon is not therapeutic inauthenticity, that is, not showing one’s true self to the therapist. Rather, deterioration was experienced within a broader experiential process beyond treatment contexts. From within the literature of psychological well-being, Ryff (2014), contributed a significant review that addresses this phenomenon, discussing the concept of eudaimonia. Here, eudaimonia refers to knowing oneself, and managing to become and to be oneself. This general process seems linked with health, both as psychological well-being, but also through inflammatory mechanisms and biological regulation (Ryff, 2014). Our findings align with such formulations, as participants describe how deterioration, on a general level, is associated with a less authentic self-relationship, as the core, and less authentic other-relationship, as a consequence. Concealing symptoms and distress from others seem part of a larger process of not being able to be, or be present with, one’s own experiences.

For helping professions, these formulations of general processes of inauthenticity as part of deterioration might have practical applications when thinking about therapeutic processes. For example, patients’ willingness to disclose thoughts, feelings, symptoms, and experiences is a necessary component of therapy and it is integral for psychotherapy to have an effect (Kleiven et al., 2020). However, many of the participants in our study expressed feeling the need to hide or suppress symptoms. In an online survey of patients’ honesty in psychotherapy, Love and Farber (2019) found that 84% of responders reported they had answered questions dishonestly about one or more topics in therapy. Such non-disclosure in therapy could negatively affect patient outcomes, and leave deterioration undetected. Notably, about one third of the responders in that study reported they believed that their non-disclosure had hurt their therapeutic progress (Love & Farber, 2019).

Lying or being dishonest are actions with strong negative connotations, and research has shown that patient deception in therapy negatively affects the therapeutic relationship and therapeutic outcomes (Hart et al., 2024). However, our results underscore a more general or broad understanding that seems more helpful in thinking about mental distress and deterioration. Also, both in our results and in previous research, patients report many reasons for concealing the truth. These include relationally oriented motives, such as wanting to be polite or not wanting to burden their therapist, as well as feeling uncomfortable with a topic or feeling embarrassed or ashamed (Blanchard & Farber, 2016; Love & Farber, 2019). Accordingly, lack of trust or an underdeveloped therapeutic alliance could explain why some patients choose to hide symptoms from their therapist (Crits-Cristoph et al., 2019; Wampold & Flückiger, 2023).

In our study, we found that participants’ concealment involved much more than a simple unwillingness to tell the truth. For some participants, concealment was even a practiced coping strategy that they had perfected over time. Such self-concealment may be perceived as useful by the individual, however, research into the concept of self-concealment shows that increased hiding and secret-keeping affects health and wellbeing, and help-seeking attitudes negatively (Larson et al., 2015). Consequently, the reluctance to disclose could be both a symptom of and risk factor for deterioration. Extensive self-concealment could lead to deterioration going unnoticed in therapy, which may deter or delay improvement. Moreover, if hiding symptoms extends to patients' answers on self-report questionnaires, these patients would not be classified as deteriorated using the standard definition of deterioration as an increase in symptoms.

Furthermore, concealment, as experienced by our participants, could also relate to a broader concept of authenticity. That suffering is related to a sense of inauthenticity (e.g., false-self theory, Winnicott, 1960/1984) has been part of psychotherapy discourse since the 1960s. Later empirical and theoretical work has shown how important authenticity is for a constructive therapeutic relationship. The real relationship (Gelso et al., 2018), an important component of the therapeutic relationship between a patient and a therapist, includes genuineness as one element. Genuineness reflects participants authenticity towards themselves and others aiming to be of help. Another element is realism - the experience or perception of the other in ways that benefit him/her. These elements are influenced by magnitude—how much genuineness and realism exists, and valence—is this realism and genuineness positive or negative (Gelso, 2014). Loss of authenticity in itself can be associated with increased suffering, and increased self-concealment by the patient can reduce the genuineness within the real relationship, affecting it negatively (Baumann & Hill, 2016). This in turn could negatively impact treatment progress and outcome.

### Deterioration in the age of measurement-based care

At the present time, monitoring patient progress and outcome by using MBC is an established practice in many healthcare systems. One important rationale for such monitoring is the potential for early detection of patient deterioration in treatment. Additionally, there is ongoing research investigating the use of patients MBC data for predicting deterioration and treatment outcome (see e.g., Lutz et al., 2025). In their study of clients’ negative experiences with treatment, Vybíral et al. (2024) highlights the need for MBC to guide therapists in identifying patient deterioration. However, the results of our study, along with previous research on patients expectations of MBC (Batterham et al., 2024; Moltu et al., 2018; van den Eshof et al., 2025), indicate that for this approach to be effective, it is essential that the instruments are designed not only to track symptom changes. For MBC to be useful, measures need to have a broad focus that seeks to capture what is important to the individual patient. The results of our study show that a central element in the experience of mental health deterioration is an overwhelming sense of depletion. MBC instruments should be designed to capture this experience, along with other functional outcomes, such as patients’ overall life functioning, quality of life, and relational aspects.

Clinically, MBC must be seen as a supplement to treatment, not as a substitute for exploring patients’ perspective. Patients must still be asked about how they experience their symptoms and overall life functioning, and how this affects their daily lives. MBC scores can be used to probe further into specific aspects of the patient’s story, and as a basis for discussion during sessions (Låver et al., 2024), but they will not capture all negative experiences or cases of deterioration.

### Implications

For MBC systems: If MBC systems are to be used for predicting deterioration, developers need a clear understanding of what this phenomenon entails. This study highlights the need for MBC to capture functional outcomes, along with the overwhelming sense of depletion patients experience when they are in deterioration. These essential components of deterioration cannot be captured by symptomatic outcome measures alone.

For clinical practice: Mental health deterioration leads to increased suffering for patients, regardless of its origin. Consequently, to adequately support the patient and maintain or strengthen the therapeutic alliance, it is important that therapists are attentive to identify whether patients are experiencing, or on the verge of experiencing, deterioration.

For training: Students must be made aware of how deterioration can manifest in patients, and that deterioration may occur independent of changes in symptoms. This awareness can lead to earlier detection of deterioration, and influence how new practitioners choose to utilise MBC data in their sessions and in session planning.

### Limitations

We interviewed patients being treated in outpatient clinics in Western Norway. As such, we were situated within the context of a Norwegian healthcare system, which differs from mental health systems in other nations. Caution should be taken when considering the generalisability of our findings to other psychotherapeutic contexts. Another limitation is the lack of racial diversity in our sample of participants. This limitation could have biased the discussion of possible influences of race and ethnicity on processes of deterioration.

For this study, we recruited participants from a general mental health and addiction clinic, without specific diagnostic inclusion criteria. Also, we only collected patients’ self-disclosed problem presentation, and not their diagnosis, and did not conduct formal assessments of participants. Thus, we are unable to specify what characterises deterioration for specific diagnostic groups. However, our results show that even though diverse, there seem to be common factors across diagnoses that define the experience of deterioration.

## Supplementary Material

Onlinesupp_distribution of subthemesvs2.docx Onlinesupp_distribution of subthemesvs2.docx

Interview guide.docx Interview guide.docx

## Data Availability

Data is available in the article. Further data will not be shared based on ethical and privacy concerns. Questions about the dataset can be directed to the first author.
