# Supplementary material for: Deterioration in mental health: towards a conceptualization based on patients’ perspectives
Source: Int J Qual Stud Health Well-being. 2026 Mar 16;21(1):2644587. doi: 10.1080/17482631.2026.2644587 (PMC12997359; doi:10.1080/17482631.2026.2644587)
Supplement: Onlinesupp_distribution of subthemesvs2.docx [file ZQHW_A_2644587_SM4062.docx]

## Online Supplement

### Distribution of subthemes in the interviews

| **Patient** | **Loosing or having one’s perspective changed** | **Being in a state of negative emotional reactivity** | **Experiencing physical and emotional pain** | **Becoming less authentic with oneself and others** |
| --- | --- | --- | --- | --- |
| **1** | x | x |  | x |
| **2** | x | x | x | x |
| **3** | x | x | x | x |
| **4** | x | x | x | x |
| **5** | x | x |  | x |
| **6** | x | x | x | x |
| **7** | x | x |  | x |
| **8** | x | x | x | x |
| **9** | x | x |  |  |
| **10** | x | x |  |  |
| **11** | x | x | x | x |
| **12** | x | x |  |  |
| **13** | x | x | x | x |
| **14** | x | x | x |  |
| **15** | x | x |  | x |
