# Supplementary material for: Deterioration in mental health: towards a conceptualization based on patients’ perspectives
Source: Int J Qual Stud Health Well-being. 2026 Mar 16;21(1):2644587. doi: 10.1080/17482631.2026.2644587 (PMC12997359; doi:10.1080/17482631.2026.2644587)
Supplement: Interview guide.docx [file ZQHW_A_2644587_SM4061.docx]

## Interview guide

1. Could you tell me a bit about your situation and why you are in treatment?
2. (While in treatment), have you ever experienced episodes of deterioration? Have you ever experienced episodes where you’re problems/condition have deteriorated?
3. How do you notice that you are deteriorating?

Prompts for probing further:

- Changes in everyday life?
- Ability to perform activities?
- Ability to do what you want to do in life?
- Social relationships and belonging?
- Feelings of inclusion/exclusion?
- Problems/deterioration hindering you/getting in the way of life?
- Thoughts about the future?

1. *If you’re therapist was really attentive, how could they recognize that you were deteriorating? What should they look for?*
2. *What do you need from treatment when you experience deterioration?*
